# A new elemental analytical approach for microplastic sum parameter analysis—ETV/ICP-MS with CO_2_

**DOI:** 10.1007/s00216-025-06146-x

**Published:** 2025-10-20

**Authors:** Vera M. Scharek, Tommy Kröger, Karin Keil, Heike Traub, Björn Meermann

**Affiliations:** 1 Federal Institute for Materials Research and Testing (BAM), Division 1.1 – Inorganic Trace Analysis (ITALab), Richard-Willstätter-Straße 11, 12489 Berlin, Germany; 2 Federal Institute for Materials Research and Testing (BAM), Division 1.9 – Chemical and Optical Sensing, Richard-Willstätter-Straße 11, 12489 Berlin, Germany

**Keywords:** Microplastics, Soil, Gas calibration, Screening, ETV/ICP-MS

## Abstract

Microplastics (MPs) are pervasive environmental pollutants and are considered one of the main challenges of our time. However, a fast and comprehensive analytical approach for MP analysis in complex matrices traceable to SI units is still lacking. In this context, we report a fast screening tool for the sum parameter analysis of MPs using electrothermal vaporization (ETV) coupled to inductively coupled plasma-mass spectrometry (ICP-MS). In our proof-of-concept study, we observed size-independent detection of MPs as peaks above the ^13^C^+^ signal background in the nano- to micrometer range without limitations regarding the polymer type. Quantification of the ^13^C^+^ MP signals was accomplished via an external gas calibration utilizing dynamic dilution of carbon dioxide with argon, yielding recovery rates of 80–96% for MP reference material (RM) of polymer types commonly found in the environment. The applicability to a soil sample was demonstrated through spiking experiments with a polyethylene (PE) MP RM in soil. The limit of detection (LOD) was estimated to be 0.13 µg C, equaling the detection of a single spherical low-density-PE particle of about 70 µm, and a limit of quantification (LOQ) of 0.42 µg C.

**Graphical abstract:**

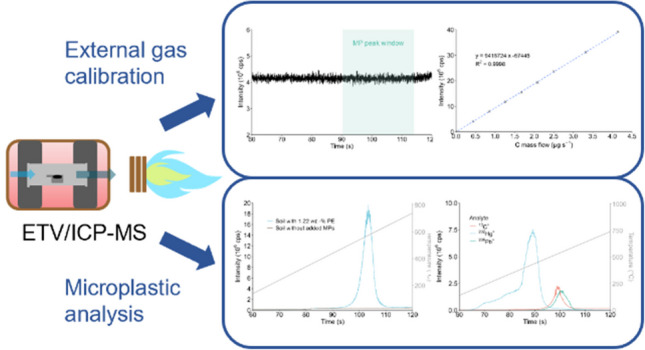

**Supplementary Information:**

The online version contains supplementary material available at 10.1007/s00216-025-06146-x.

## Introduction

Global plastic pollution is considered one of the biggest challenges of our time, mainly attributed to the mismanagement of plastic waste entering the oceans or contaminating terrestrial ecosystems [1]. Through weathering processes, microplastics (MPs) are formed [2] or released due to mechanical stress (e.g., tire abrasion and synthetic clothing wear) [3, 4]. On a global scale, 1.5 to 6 Mt of MPs are estimated to be in soil alone [5]. A harmonized definition of MPs is still lacking [6]. The term “microplastics” mostly refers to solid, persistent, synthetic polymer particles of any shape with a size < 5 mm, while particles smaller than 1 µm are designated as nanoplastics (NPs) [6]. In contrast to macroplastics (> 5 mm), toxicological concerns mainly arise from the small size of MPs, leading to their uptake and accumulation [7–9]. As a result, MPs have been detected in numerous organisms and were found in diverse human organs and body fluids [8–10]. Hereby, MPs can act as a transport vector for hazardous additives [11] and contaminants [12] and are suspected to induce oxidative stress and inflammatory responses [8].

Numerous studies have been conducted to assess the pollution situation. However, the lack of harmonized methods for quantifying MPs has led to incomparable data [13]. Data acquired by spectroscopic approaches, such as Fourier transform infrared (FTIR) [14, 15], and Raman spectroscopy [16], are based on particle number per sample mass and limited to a certain size fraction depending on the applied sample preparation and analytical approach [14]. Several authors highlight the need for standardized analytical methods traceable to SI units regarding regulatory limits and pollution monitoring [17–19]. The quantification of MPs based on their mass is achievable through thermoanalytical methods, where the polymer material undergoes thermal decomposition. MPs in environmental samples are commonly determined by pyrolysis-gas chromatography-mass spectrometry (Py-GC-MS) and thermoextraction and desorption-gas chromatography-mass spectrometry (TED-GC-MS), which enable the identification and quantification of polymers based on their decomposition products [18, 20, 21]. However, quantitative data are polymer-specific, and method development is often only carried out for high-abundant polymers while other types remain undetected. Additionally, low sample masses in the case of Py-GC-MS and interfering matrix components that cannot be chromatographically separated require laborious sample preparation procedures [19].

Recently, inductively coupled plasma-mass spectrometry (ICP-MS) in single-particle (sp-ICP-MS) mode has gained popularity for the characterization and quantification of MPs via carbon isotope detection [22–25]. Apart from this, carbon is mainly utilized for its signal enhancement effect on certain elements such as As, Se, and Te in ICP-MS measurements [26]. Few applications for analyzing carbon directly via ICP-MS have been reported [27–29]. Carbon is poorly ionized in the ICP (1–5%) due to its high ionization energy (IE = 11.26 eV) and space charge effects, leading to lower sensitivities compared to the analysis of metals [29]. Additionally, high background intensities derived from dissolved CO_2_in nebulized samples and impurities in argon gas, as well as the atmosphere, respectively, are reported [27, 29]. Concerning its low abundance, the ^13^C isotope (~ 1% of ^12^C) is often acquired to reduce matrix-related signal intensities despite the higher sensitivity achieved by the ^12^C isotope [24, 25]. The size range of detectable MP particles with reported 0.62 µm (in ultra-pure water) [22] up to 5–6 µm [24] is limited by sensitivity at the lower as well as sample introduction and complete ionization at the upper limit, respectively. Consequently, MPs are not fully captured regarding the common MP definition (1–5000 µm), and the application to complex environmental matrices remains difficult. So far, analyzing MPs via sp-ICP-MS has been applied to, e.g., suspended consumer products [24], seawater [22], and via sp-laser ablation-ICP-MS to artificial soil [30].

Reported calibration approaches for the ICP-MS response of carbon are based on dissolved organic substances, such as tartaric acid [24], or glucose [31]. A new gas calibration technique with carbon dioxide has been implemented for determining polystyrene (PS) particles in a simulated seawater matrix by Mervic et al*. *[25]. The procedure originally developed for a matrix-matched calibration and high reproducibility for single MP ICP-MS shows potential for the application of dry aerosol systems (e.g., solid sampling techniques) as stated by the authors [25]. By enabling the direct introduction of solid samples, techniques such as electrothermal vaporization (ETV) benefit from reducing laborious, chemical, and time-consuming sample preparation procedures while being less prone to errors originating from contamination and analyte loss, respectively. In principle, the homogenized sample is placed on a sample holder and transferred into the ETV furnace, where it is resistively heated under an argon atmosphere, and transported into the ICP via an argon stream. ETV/ICP-MS methods have been developed for environmental analysis, e.g., mercury in sludge samples [32] or platinum and rhodium in tunnel dust [33], and applied to polymer matrices [34, 35]. Young and Jones determined the total carbon content of soft drinks by ETV/ICP-atomic emission spectroscopy of the tungsten coil type, where a tungsten filament is used as a vaporizer in contrast to commercially available ETV units of the graphite tube type [36]. However, to the best of the authors’ knowledge, ETV has not been utilized to directly determine MPs so far.

This work aimed to develop an ETV/ICP-MS-based fast screening method for a comprehensive MP analysis in a soil sample. In our proof-of-concept study, we mainly focused on (i) evaluating the applicability of ETV as a pyrolysis unit for the sum parameter analysis of MPs, (ii) the implementation and validation of a gas calibration method for a new mass balance approach, and (iii) proving the applicability of the approach for a soil matrix. Method development and validation were preferably carried out with MP reference materials (RMs) and RM candidates to ensure conditions close to reality. Thereby, polyethylene (PE), polypropylene (PP), and polyethylene terephthalate (PET) were chosen because of their high abundance in the environment [14, 15]. Particularly, PE is reported to be the most common polymer type in soil [14, 15]. As a real-world matrix in spiking experiments, standardized soil was utilized, which has been used as a “MP-free” matrix in the literature before [17, 37].

## Experimental

### Materials

MP RMs BAM-P201 [38] and BAM-P206 [39], and a RM candidate (BAM-P208) from BAM (Federal Institute for Materials Research and Testing, Berlin, Germany) were used for method development and validation. Carbon mass fractions of the polymer materials were verified using elementary analysis (EuroVector Elemental Analyser, Pavia, Italy) (Table 1).

A PS particle size standard PS-ST-400.0 (2 wt.% aqueous suspension) with a certified diameter of 390 µm was obtained from microParticles GmbH (Berlin, Germany), and carboxylated PS microparticles PPS-0.1 (B version) (2.5 wt.% aqueous suspension, *d* = 0.1 µm) from Kisker Biotech GmbH & Co. KG (Steinfurt, Germany). Particle suspensions were further diluted with water purified by a Milli-Q water purification system (Merck Millipore, France) to a concentration of 10 g L^−1^ based on the particle mass. Polyether sulfone (PES) granulate from BASF (Ludwigshafen, Germany) was provided by the University of Applied Science Münster (Steinfurt, Germany). Acrylonitrile butadiene styrene (ABS) certified reference material (CRM) BAM-H010 granulate with mass fractions of (479 ± 17) µg g^−1^ for lead (*k* = 2) and 415 µg g^−1^ for mercury (informative value) was obtained from BAM (Berlin, Germany) [40]. PES and ABS MP materials, respectively, were prepared using a centrifugal mill (ZM 200, Retsch, Haan, Germany) equipped with a stainless-steel ring sieve with trapezoidal holes of 1.00 mm after pre-cooling the granulate with liquid nitrogen. Polyvinyl chloride (PVC) powder of high molecular weight was purchased from Sigma-Aldrich (Steinheim, Germany). Standard soil LUFA 2.3 (LUFA Speyer, Speyer, Germany) of the type sandy loam (soil type according to United States Department of Agriculture) with an organic content of 0.75 ± 0.11% C (w/w) was used for spiking experiments.

**Table 1:** Characteristics of MP RMs and RM candidates

Art. no	Description	Particle size D50^a^ (µm)	Mass fraction of C^b^ (%)	Mass fraction of C^c^ (RSD, %), *n* = 3	Recovery^d^ (%)
BAM-P201	Cryo-milled, artificially aged PE foil	61.2	85.6	86.0 (0.3)	100.5
BAM-P206	Milled PET granulate in bottle-grade quality	62.5	62.5	62.6 (0.9)	100.2
BAM-P208	PP powder	262	85.6	85.9 (0.3)	100.4

^a^Determined by laser diffraction under dry dispersion

^b^Calculated from the molar mass of carbon and the molar mass of the repeating unit

^c^Mass fraction and relative standard deviation (RSD) determined by elementary analysis

^d^Calculated as the quotient of the measured and theoretical mass fraction of C

### Instrumentation

For ETV/ICP-MS measurements, a commercially available ETV system of the graphite tube type ETV-4000d equipped with an autosampler AD30 from Spectral Systems (Fürstenfeldbruck, Germany) was coupled to a quadrupole-based ICP-MS Thermo Scientific iCAP Qc (Thermo Fisher Scientific, Bremen, Germany). A quartz torch and injector pipe (2.5-mm inner diameter) and an alumina sampler and nickel skimmer cone for high matrix from Glass Expansion (Weilburg, Germany) were used. Detailed operating information is given in Table 2.

**Table 2:** Operating and acquisition conditions of the ETV/ICP-MS system

Parameter	Value
ICP-MS instrument	iCAP Qc
Cones	Al sampler cone, Ni skimmer cone for high matrix
Plasma power (W)	1500
ICP gas flows (L Ar min^−1^)	0.65 (auxiliary gas), 14 (cool gas)
Sampling depth (mm)	5.0
ETV system	ETV-4000d equipped with autosampler AD30
Sample boats and tubes	Pyrolytically coated graphite, size “maxi”
ETV gas flows (L Ar min^−1^)	0.142 (carrier gas), 0.420 (bypass gas)
Isotopes monitored	^13^C^+^, [^40^Ar]_2_^+^, ^26^ Mg^+^ for soil analysis, ^35^Cl^+^ for PVC analysis, ^202^Hg^+^ and ^208^Pb^+^ for additive analysis with 10 ms dwell time in time-resolved mode

Prior to each measurement day, the temperature of the internal ETV pyrometer was compared and corrected using an external pyrometer PKL 38 AF 1 (KELLER HCW GmbH, Ibbenbüren, Germany). Pyrolytically coated sample boats and tubes of the size “maxi” were purchased from Spectral Systems (Fürstenfeldbruck, Germany). ETV flow rates of the carrier and bypass gas (0.142 L min^−1^ and 0.420 L min^−1^ argon, respectively) were applied as recommended by the manufacturer.

For solid sample introduction, MP samples of 6.0 µg–20 µg and soil material of (1.0 ± 0.1) mg, respectively, were weighed into pyrolytically coated graphite boats and automatically transferred into the ETV furnace. A UMT5 microbalance (*d* = 0.0001 mg, min. 0.01 mg) from Mettler-Toledo GmbH (Gießen, Germany) was used. For spiking experiments, samples of ~ 2 wt.% PE RM in soil material were prepared by adding about 1 mg of soil material on top of BAM-P201 in graphite boats. Prior to analysis, aqueous PS particle suspensions (1–10 µL of 10 g L^−1^ suspension) were dried in a graphite boat using an infrared drying device (Spectral Systems, Fürstenfeldbruck, Germany). After a pre-heating step to condition the ETV system at 80 °C for 20 s, the temperature was increased from 100 to 800 °C with 10 K s^−1^ to ensure consistent pyrolysis of the polymer material. During the ETV program, all isotopes were monitored in time-resolved mode with 10 ms dwell times via ICP-MS. ^13^C^+^ was acquired for carbon analysis and [^40^Ar]_2_^+^as an indicator of plasma loading effects [41]. For the analysis of soil samples, ^26^ Mg^+^ was monitored to ensure the absence of the double-charged ^26^ Mg^++^ interference.

### Gas calibration

**Fig. 1:**
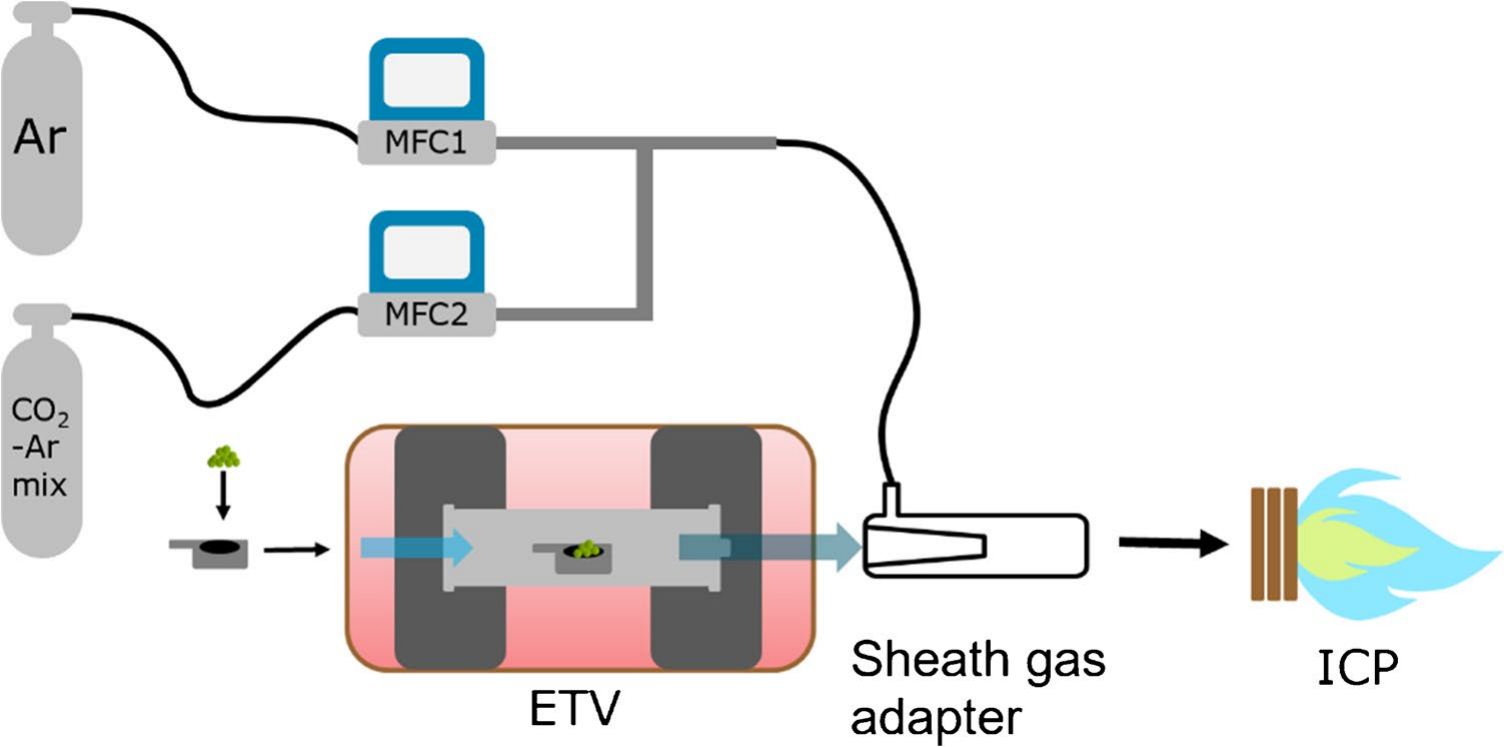
Scheme of the gas calibration setup coupled to ETV/ICP-MS

A primary reference gas mixture with 1.1% carbon dioxide in argon (CO_2_ (1.104347 ± 0.003) % (w/w), Ar (98.895599 ± 0.001) % (w/w), expanded uncertainties with *k* = 2) produced according to ISO 6142 [42] by BAM (Berlin, Germany) was dynamically diluted with Ar gas in the quality grade 5.0 (Linde GmbH, Pullach, Germany) as described by Mervic et al. [25]. A scheme of the instrumental gas calibration setup coupled to ETV/ICP-MS is shown in Fig. 1. Ar-calibrated thermic mass flow controllers (MFCs) EL-FLOW® Select F-201CV (flow ranges max. 50 mLn min^−1^ for the CO_2_-Ar gas mixture and 500 mLn min^−1^for the diluting Ar) equipped with BRIGHT B2 IP40 display and control modules from Bronkhorst GmbH (Kamen, Germany) were utilized for controlling and adjusting the gas flow rates. The calibration of the MFCs was performed upstream with laminar flow elements Molbloc L 5E2 and Molbloc L 5E1 (Fluke Calibration, Everett, WA) [25]. Merging of the reference and diluting gas flows was accomplished via stainless-steel pipes in a T formation. A total gas flow rate of 500 mL min^−1^ was maintained throughout the entire analysis to ensure consistent plasma conditions. A sheath gas adapter purchased from AHF (Tübingen, Germany) was inserted before the ICP torch to allow the concurrent introduction/mixing of the ETV and calibration gas flows. Gas calibration measurements were conducted alongside ETV measurements of an empty graphite boat to ensure conditions as present for subsequent solid sample measurements. Flow levels of the reference gas mixture were selected to encompass the intensity range of the MP analyte peak (typically up to 50 mL min^−1^).

### Data processing

The raw data files exported from the ICP-MS software Qtegra (Thermo Fisher Scientific, Bremen, Germany) were processed using the programming language *R*. The workflow can be divided into five steps: calculating the carbon mass flow, setting up the linear calibration equation, converting the ETV time scans, baseline correction, and peak integration. The gas flow of the CO_2_-Ar reference gas mixture *Q*_mix_ in mL min^−1^ was converted to the carbon mass flow (${\dot{m}}_{C}$) in µg s^−1^ based on Eq. 1.

1 $${\dot{m}}_{\mathrm{C}}= {Q}_{\mathrm{mix}}\bullet {\rho }_{\mathrm{mix}}\bullet {w}_{{\mathrm{CO}}_{2}}\bullet {w}_{\mathrm{C}}\bullet \frac{1000}{60}$$

The mass fraction of CO_2_ in the reference gas mixture ${w}_{{\mathrm{CO}}_{2}}$ was taken from the certificate of the primary reference gas mixture, and ${w}_{\mathrm{C}}$ is the mass fraction of carbon in carbon dioxide. The density of the Ar-CO_2_ gas mixture ${\rho }_{\mathrm{mix}}$ at room temperature was calculated after the ideal gas law equation in g L^−1^ (SI, Eq. S1). Mass flows of different CO_2_ levels were plotted against the respective mean ^13^C^+^ signal intensities in the expected MP peak windows to obtain the linear calibration equation. Intensities of the ETV/ICP-MS time scan were converted point-by-point to the corresponding mass flows via the calibration equation to determine the polymer mass in % C. Subsequent integration of the baseline-corrected mass flow diagram provided the absolute carbon amount in micrograms. For validation purposes, the absolute polymer mass was calculated based on the mass fraction of C in % of the respective polymer types.

The limits of detection (LOD) and quantification (LOQ) were estimated as three and ten times, respectively, the standard deviation of soil measurements (*n* = 9) without added MPs in the MP peak window ${s}_{\mathrm{L}}$ (in counts) divided by the slope of the linear gas calibration equation *b* (counts µg^−1^). Additionally, the size detection limit (sDL) in micrometers as the minimum detectable diameter of a single PE particle in soil was evaluated under the assumption of a spherical low-density-PE (LDPE; ${\rho }_{\mathrm{LDPE}}$ = 0.915 g cm^−3^) particle based on the LOD in ng as follows—Eq. 2:

2 $$sDL= \sqrt[3]{\frac{6000 \bullet \mathrm{LOD}}{{\rho }_{\mathrm{LDPE}}\bullet\uppi }}$$

## Results and discussion

### ETV method development

**Fig. 2:**
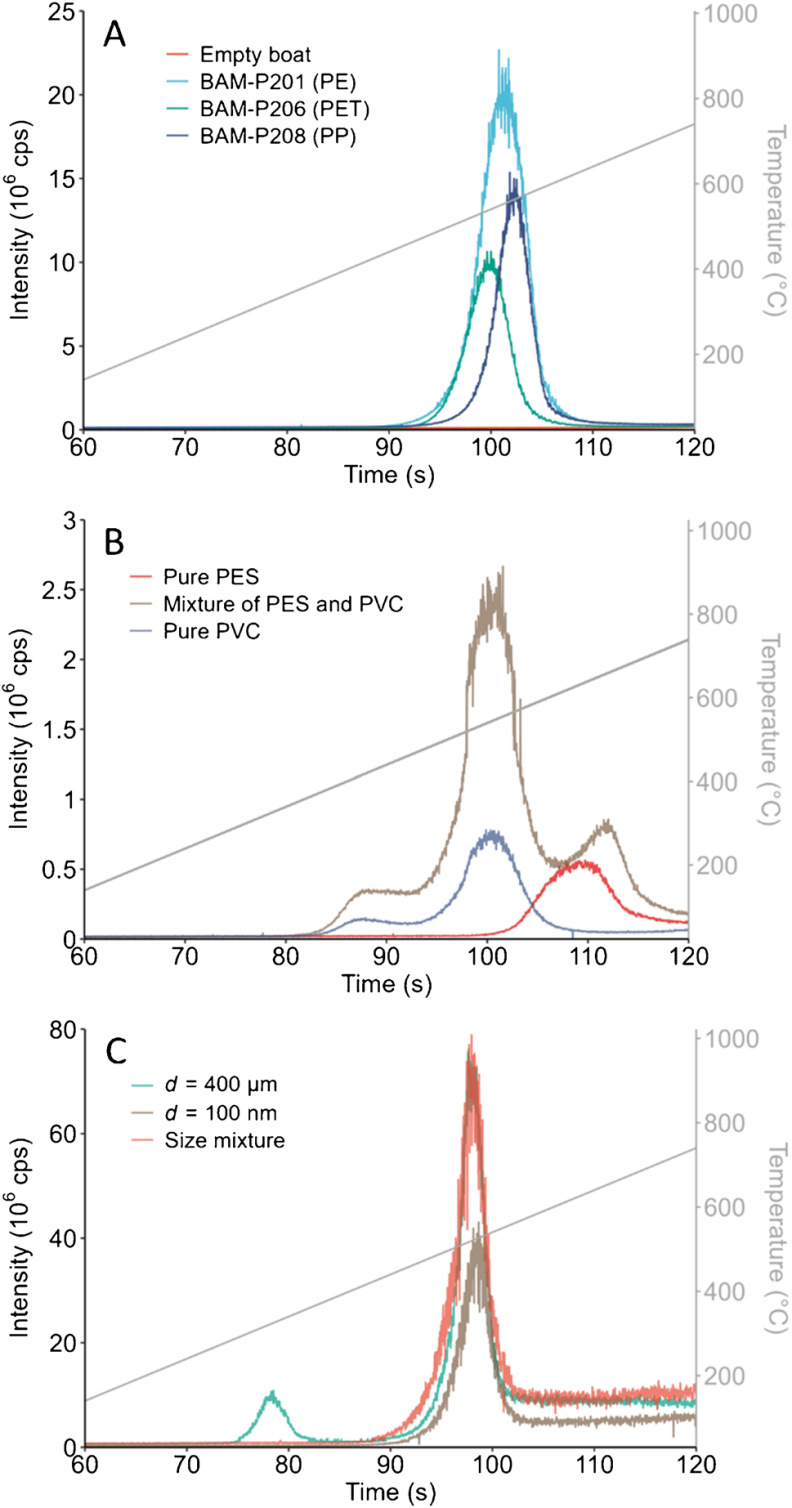
^13^C^+^ time scans of the ETV/ICP-MS measurement of MP material. The temperature program is shown in gray. **A** The MP RMs BAM-P201 (PE, *m*_PE_ = 8.1 µg C), BAM-P206 (PET, *m*_PET_ = 6.6 µg C), BAM-P208 (PP, *m*_PP_ = 7.4 µg C), and an empty graphite boat were measured. **B** Pure PES (*m*_PES_ = 10.6 µg C), PVC (*m*_PVC_ = 6.0 µg C), and the mixture of both polymers (*m*_PES_ = 14.8 µg C, *m*_PVC_ = 13.2 µg C) were measured. **C** PS particles of 100 µm (*m*_100 µm_ = 11.5 µg C) and 400 µm (five particles) and a mixture of these particles (*m*_100 µm_ = 11.5 µg C, one 400 µm particle) were measured

ETV in coupling with ICP-MS has the potential as a fast screening tool for the sum parameter analysis of MPs. However, the furnace interior of commercially available ETV systems is built out of graphite material and undergoes thermal wear during analysis. To investigate the analytical capability of a graphite tube ETV/ICP-MS system for MP detection, MP RMs were directly placed into a graphite boat and analyzed alongside an empty graphite boat. As common polymer types such as PP, PE, and PS exhibit pyrolysis temperatures ranging from ~ 390 to 490 °C, the applied ETV program encompassed 100–800 °C [43]. Similar temperature ranges are used in thermogravimetric analysis (TGA; 100–900 °C) [20, 37]. Excerpts of the time-resolved ^13^C^+^ intensity data are shown in Fig. 2. The less abundant ^13^C isotope was recorded exclusively due to high ^13^C^+^ signal intensities. With a temperature ramp of 10 K s^−1^, the ^13^C^+^ background signal of the system remained relatively consistent up to an applied temperature of ~ 700 °C (Fig. S1 in the SI), while MP materials were detected as peaks (Fig. 2A). The ^13^C^+^ background signal in the MP peak window was equivalent to a carbon content of 0.53 µg. All three RMs were detected within the same time window (equalling pyrolysis temperatures of about 440–590 °C calculated based on the temperature program and detection time) at about 100 K higher than the reported pyrolysis temperatures of the respective polymer types [43, 44]. This could be attributed to the heating duration of the ETV system and the transfer time from ETV to ICP-MS. Since the main aim was to develop a method for the sum parameter analysis of MPs, the applicability of the ETV method for polymer types of a wider pyrolysis temperature range was investigated. For the low pyrolysis temperature range, PVC and a high-temperature pyrolyzing polymer PES, respectively, were selected. ^13^C^+^ signals of the polymer mixture were detected at about 340–740 °C, originating from PVC (340–600 °C) and PES (600–740 °C) (Fig. 2B). The detection of a double peak for PVC is also reported in the literature for TGA measurements and assigned to the two-step pyrolysis mechanism of the polymer material [43]. Further optimization could include optimizing the ETV temperature program to unite the polymer peaks for improved sensitivity.

With BAM-P201, BAM-P206, and BAM-P208, materials of a similar size range (D50 ~ 60 µm and 262 µm, respectively) were analyzed (Fig. 2A). To further evaluate the influence of the particle size on the decomposition via ETV, diluted suspensions of PS particles in the nanometer (*d* = 100 nm) and micrometer (*d* = 390 µm) range were measured individually and in a mixture by ETV/ICP-MS. PS particles were utilized as they are available in well-characterized sizes. Respective PS particle peaks were detected in the 430–590 °C temperature range as observed for the RMs (Fig. 2C). A smaller ^13^C^+^ peak at 80 s (340 °C) can possibly be explained by non-polymeric organic content, e.g., surfactants, which are reported to be used for particle stabilization [45]. Decomposition of the particle size mixture resulted in one peak. A size fractionation was not visible. The results show that the developed ETV/ICP-MS method is suitable for the analysis of MPs and MP mixtures of different polymer types and sizes.

### Validation of the method with different MP RMs

**Fig. 3:**
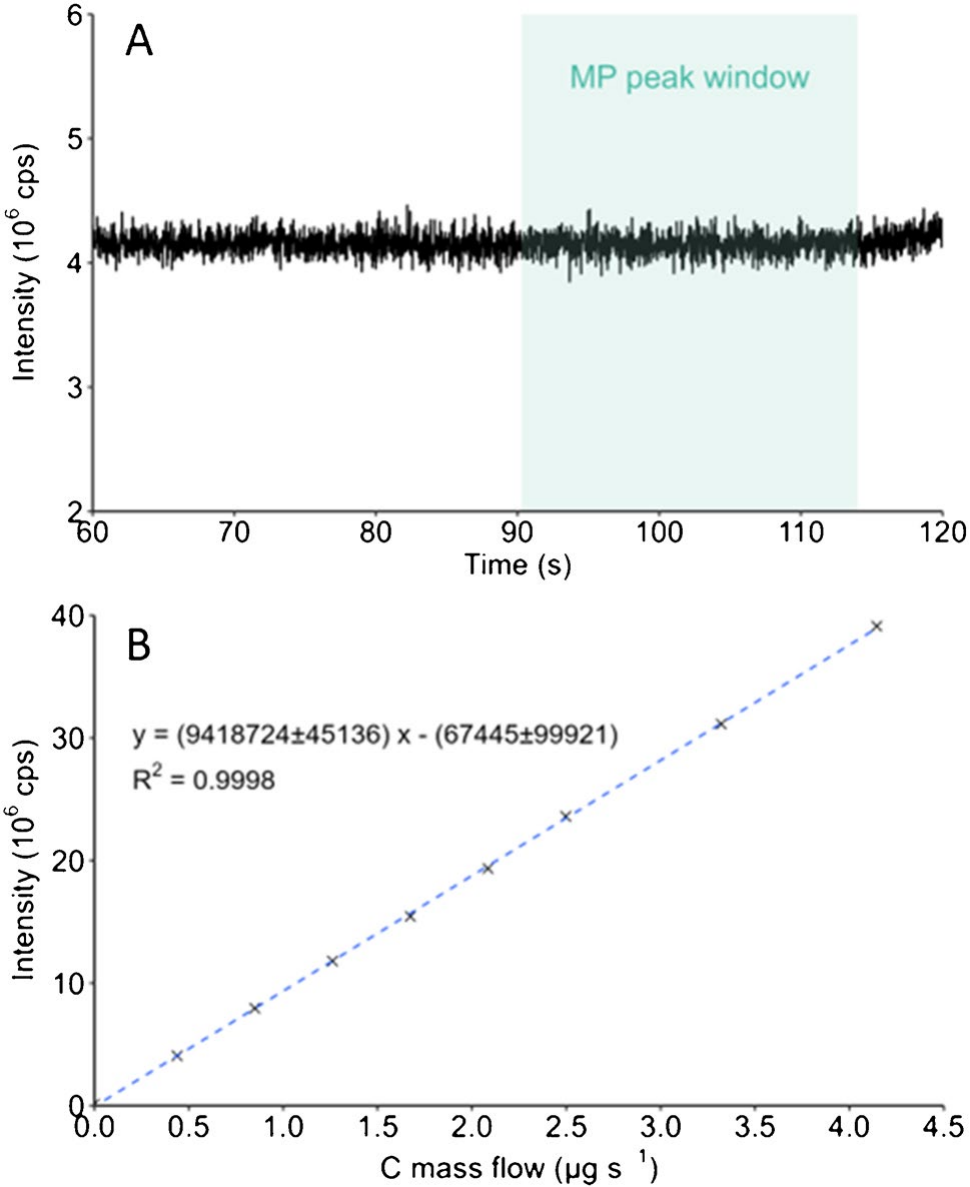
**A** Example ETV/ICP-MS time scan of a calibration measurement with an introduced CO_2_ gas flow of 0.87 µL s^−1^. The time range used to calculate the mean ^13^C^+^ signal intensities is marked in green. **B** Mean ^13^C^+^ signal intensities as a function of the carbon mass flow for determining the calibration equation

Conventional calibration approaches for ETV include external calibration with dried aqueous standards [34, 35], solid RMs [32], and standard addition [33, 36]. However, regarding MP materials, these methods are limited since (i) preparation and dilution of aqueous suspensions are not feasible due to floating or sedimentation of the material depending on its density; (ii) preparation and dilution of solid mixtures, e.g., with matrix material, suffers from low homogeneity due to the particulate nature of the material; and (iii) precise weighing of required masses (low µg-range) remains difficult. Here, an alternative gas calibration approach was established for ETV/ICP-MS, which was recently developed for single MP ICP-MS analysis independent of the matrix and MP type [25]. Comparable plasma conditions throughout the sample and calibration measurements were ensured by (i) maintaining the total gas flow, (ii) ETV runs of empty sample boats during calibration measurements, and (iii) evaluating signal intensities in the MPs’ peak window. Gas mixtures of at least five different CO_2_ portions were measured, yielding a stable ^13^C^+^ signal in the MP peak window (Fig. 3A). The mean intensity values as a function of the carbon mass flows are given in Fig. 3B. A linear correlation with *R*^2^ ≥ 0.9995 was found on different measurement days.

To investigate the applicability of the external gas calibration for the quantification of MPs with different polymer types, the MP RMs BAM-P201, BAM-P206, and BAM-P208 were used for method validation. Recoveries were calculated as the ratio between the determined carbon mass and the theoretical carbon content of the material, yielding 80.4–96.3% for the respective polymer types (Table 3). A recovery of 96.3% for PE indicates a transport efficiency of almost 100%, on the supposition that the CO_2_-Ar gas mixture is completely transported into the ICP. Relative standard deviations (RSDs) for the triplicate measurements were consistently below 5%, demonstrating the reproducibility of the ETV-ICP-MS approach. The lowest recovery determined for PET, at 80.4%, could result from residual analyte in the graphite boat after the analysis. For PET, a charred residue is reported to account for about 9% of the starting mass in TGA, while PE fully volatilizes [19]. The argon dimer did not show changes in the signal intensities during sample pyrolysis that would explain the recoveries of PET and PP by suppression of the ^13^C^+^ signal.

**Table 3:** Validation results of the ETV/ICP-MS measurement of MP RMs (*n = 3*)

MP material	Polymer mass^a^ (µg C)	Polymer mass^a^ (µg)	Recovery (RSD, %)
PE (BAM-P201)	8.0 (8.3)	9.4 (9.7)	96.3 (4.8)
PP (BAM-P208)^b^	7.1 (8.4)	8.3 (9.8)	84.8 (4.0)
PET (BAM-P206)	8.0 (9.9)	12.9 (15.8)	80.4 (4.6)

^a^Determined values are listed with the mean weighted portions and equaling carbon masses in brackets

^b^Here: *n* = 2

### Quantification of PE in a soil matrix

To assess the feasibility of the ETV method for real-world samples, standard soil LUFA 2.3 was utilized as an environmental matrix. Uncontaminated environmental samples are rare since MPs are widely distributed and pervasive in the environment [14, 15]. However, the soil used in this study has been well characterized by Paul et al. [37] regarding MP contamination and used as a “MP-free” matrix [17, 37]. We observed an increase in the ^13^C^+^ signal at about 340 °C for ETV/ICP-MS measurements of pure soil compared to blank measurements of empty graphite boats, indicating the pyrolysis of organic soil components (SI, Fig. S2). The ^13^C^+^ signal intensity proceeded relatively constant at the pyrolysis temperature range of the MP RMs (440–590 °C) until a further increase at ~640 °C. ^13^C^+^ signal intensities varied between different measurement runs, potentially due to the inhomogeneity of the sample. However, background signal intensities were equivalent to a carbon content of less than 1.9 µg. The absence of interference from double-charged ^26^ Mg^++^ on ^13^C^+^ was confirmed by monitoring the ^26^ Mg isotope. Interferences from ^1^H^12^C^+^ were not expected due to the dry aerosol conditions.

**Fig. 4:**
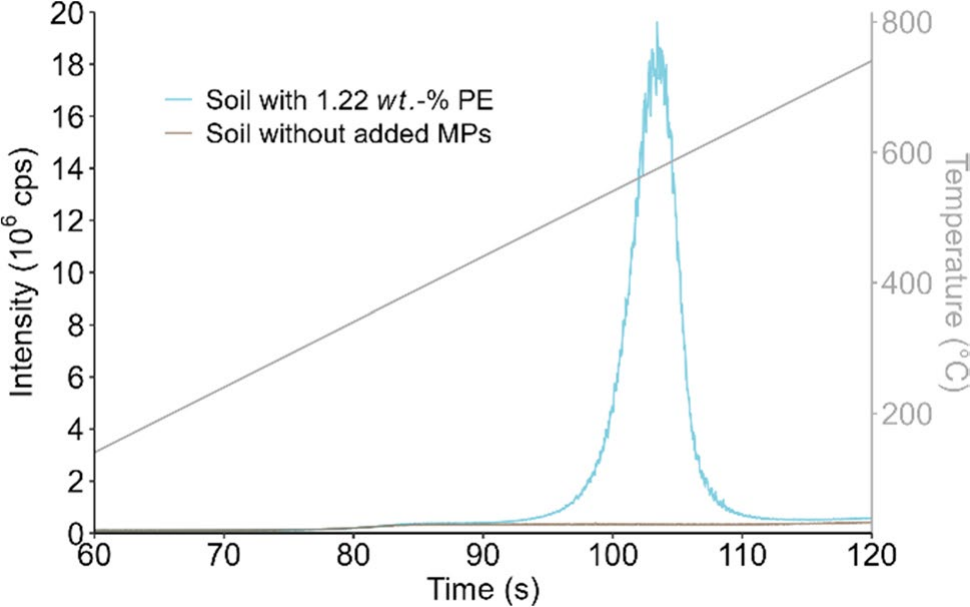
^13^C^+^ time scans of the ETV/ICP-MS measurements from spiking experiments with soil. PE in soil (*w* = 1.22 wt.%) and soil without added MPs with a mass of about 1 mg were measured. The temperature program is shown in gray

PE was selected for validation with a soil matrix as it is reported to be one of the most abundant MP materials in terrestrial ecosystems [14, 15]. Spiking experiments were carried out by weighing the MP material into graphite boats and covering it with soil matrix, which allowed for a precise adjustment of the MP content in the matrix while ensuring contact of MPs and soil compounds. Substantial MP-matrix interactions, which would either hinder or promote the volatilization and transport of the pyrolysis products, were not expected by this procedure. By covering the MP material with soil, the polymer pyrolysis products could be physically retained. However, we did not observe a delay of the MP peak. For a PE content of 1.22 wt.%, the ^13^C^+^ signal of the MP peak height was multiple times above the signal originating from the soil (Fig. 4). The decomposition of the PE MPs was observed at about the same temperature range as for the pure material. Using the developed CO_2_ gas calibration approach, the average recovery yielded 91.2% (RSD = 3.2%, *n* = 3) for ~ 2 wt.% PE in about 1 mg soil, comparable to the determined value for the pure PE material (recovery 96.3%). The estimated LOD and LOQ are shown in Table 4. Absolute and concentration values based on the carbon and PE content, respectively, are given for comparison. For PE, a LOD of 0.15 µg was estimated to be equivalent to a single 0 of about 70 µm (sDL). With 150 µg g^−1^, the LOD for PE was 30 times higher than the reported method detection limit of 5 µg g^−1^for a Py-GC-MS approach [20]. This can be explained mostly by the 4000 times higher sample mass (4 g) achieved through an extraction procedure prior to GC analysis. A theoretical LOQ was given with 0.2 wt.% for TED-GC-MS estimated for a signal-to-noise ratio of 6.18. However, as stated by the authors, concentrations lower than 1 wt.% were not evaluable.

### Polymer characterization via heteroatoms

**Fig. 5:**
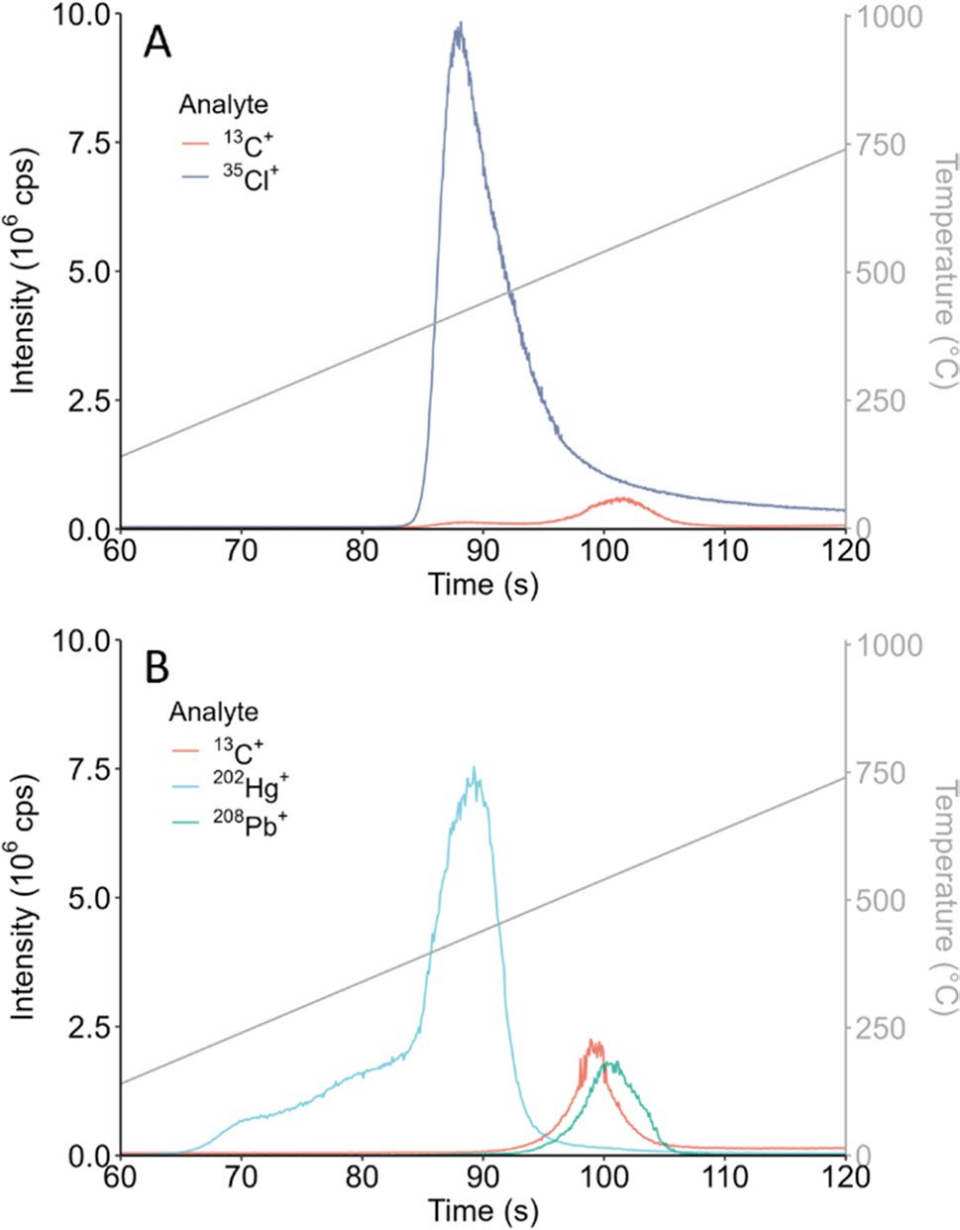
Multi-element ETV/ICP-MS time scans. **A** PVC (*m*_PVC_ = 12.4 µg) and **B** milled ABS material (*m*_ABS_ = 15.6 µg) were measured. The temperature program is shown in gray

In contrast to sophisticated techniques such as Py-GC-MS, no structural information on the analyte is received via ETV/ICP-MS measurements. However, ICP-MS has the advantage of multi-elemental acquisition and, hence, the potential to identify heteroatom-containing polymers, e.g., Cl in PVC. As shown in Fig. 5A, the ^35^Cl^+^ signal peak aligns with the first ^13^C^+^signal peak for the ETV/ICP-MS measurement of PVC MP material, presumably due to the release of chlorine-containing pyrolysis products in the first decomposition step [43]. The signal height of the Cl peak is notably higher (~ 16 times) than that of the carbon peak, opening the possibility for a sensitive and polymer-specific MP determination.

**Table 4:** Validation results of the ETV/ICP-MS measurement of MP RMs (*n = 3*)

Figure of merit	LOD	LOQ
Absolute carbon mass (µg)	0.13	0.42
Absolute PE mass (µg)	0.15	0.50
Carbon mass concentration^1^ (µg g^−1^)	130	420
PE mass concentration^1^ (µg g^−1^)	150	500

Additionally, various additives of inorganic nature, e.g., inorganic fillers, or with heteroatoms, such as brominated flame retardants, are present in plastics [46]. Milled ABS CRM, containing mercury oxide and lead stearate, was used to evaluate the capability of our method for the analysis of plastic additives. With the applied ETV temperature program, a broad ^202^Hg^+^ signal peak was observed mainly before the ^13^C^+^ signal peak, whereas a lead peak was detected slightly offset to higher furnace temperatures (Fig. 5B). Unlike Cl in PVC, where the analyte co-vaporized with the polymer material, the additive detection time appears to be less dependent on the decomposition temperature of the polymer. Instead, it seems to be more influenced by the individual boiling points of the substances and interactions with the polymer. Given the (eco-)toxicological significance of polymer additives, analyzing MPs along with their additives presents new opportunities for assessing the risks associated with MPs.

## Conclusions

Our proof-of-concept study demonstrates the potential of ETV/ICP-MS as a fast screening tool for the sum parameter analysis of MPs in a soil sample. The feasibility of ETV as a solid sampling system for MPs was assessed using three MP RMs (PE, PP, PET), representing common polymer types in the terrestrial environment. In addition, PVC and PES MP materials were analyzed to evaluate the method’s applicability for a wider range of pyrolysis temperatures, while the influence of the particle size was investigated using PS particles in the nano- and micrometer-size range. Traceability to SI units was achieved by calibrating the transient ^13^C^+^ signal peaks via an external gas calibration approach. Here, different levels of carbon mass flows were introduced into the ICP-MS through dynamic dilution of a carbon dioxide–containing primary reference gas with argon. To confirm the applicability for a soil sample, PE was quantified in a well-characterized “MP-free” soil matrix after MP spiking.

We showed that MPs are directly detectable by ETV/ICP-MS as peaks above a relatively stable carbon background between 440 and 590 °C (PE, PP, and PET). The MP peak window was slightly expanded using material pyrolyzing at lower and higher temperatures. We did not observe a recognizable size-dependent influence. The external gas calibration approach yielded calibration curves of high linearity (*R*^2^ ≥ 0.9995) and enabled the successful quantification of the pure MP RMs (recovery80.4–96.3%). We observed an elevation of the carbon background for soil samples with an organic carbon content of 0.75% C. However, the ^13^C^+^ background signal remained relatively stable in the MP peak window, allowing the separation of the MP peak from the matrix background signal via baseline correction with a linear spline. The recovery of the PE MP RM in the soil matrix yielded 91.2%. In addition, we showed that the multi-elemental capability of the ICP-MS enables the sensitive detection of PVC by measuring ^35^Cl^+^ and the acquisition of metal-based polymer additives.

It should be noted that identification of the polymer types and respective portions is necessary for calculating the polymer content. However, our method allows for a fast, mass-based assessment of the pollution situation without limitations based on size or polymer type, making it a valuable complementary approach to existing analytical tools. In a multi-elemental approach, polymer types could be determined by their incorporated heteroatoms. Monitoring additives, e.g., brominated flame retardants or metal-containing stabilizers, could serve as elemental fingerprints and assist in the risk assessment of real-world samples [45]. Further research is required to determine if the method can be applied to soils and other environmental matrices with higher organic carbon content. If needed, actions should be taken to reduce the ^13^C^+^ signal background or to separate interfering matrix components by adjusting the ETV temperature program. In this context, the availability of a well-characterized matrix is crucial to distinguish synthetic particles from organic matrix components.

## Supplementary Information

Below is the link to the electronic supplementary material.

Supplementary file1 (DOCX 79.2 KB)

## Data Availability

All data will be made available upon request to the corresponding author.
