# Supplementary material for: A new elemental analytical approach for microplastic sum parameter analysis—ETV/ICP-MS with CO2
Source: Anal Bioanal Chem. 2025 Oct 20;418(16):5133–42. doi: 10.1007/s00216-025-06146-x (PMC13423920; doi:10.1007/s00216-025-06146-x)
Supplement: Supplementary file 1 — Supplementary file1 (DOCX 79.2 KB) [file 216_2025_6146_MOESM1_ESM.docx]

Supporting Information

A new elemental analytical approach for microplastic sum parameter analysis—ETV/ICP-MS with CO_2_

Vera M. Scharek^1^, Thommy Kröger^1^, Karin Keil^2^, Heike Traub^1^, Björn Meermann^1^*

^1^Federal Institute for Materials Research and Testing (BAM), Division 1.1 – Inorganic Trace Analysis (ITALab), Richard-Willstätter-Str. 11, 12489 Berlin, Germany

^2^Federal Institute for Materials Research and Testing (BAM), Division 1.9 – Chemical and Optical Sensing, Richard-Willstätter-Str. 11, 12489 Berlin, Germany

Corresponding author: PD Dr. habil. Björn Meermann, [bjoern.meermann@bam.de](mailto:bjoern.meermann@bam.de)

The Ar-CO_2_ gas mixture $\rho_{mix}$ at room temperature was obtained on basis of the ideal gas law equation using Eq. S1. For the pressure *P*, the atmospheric value of 101,325 Pa was utilized. *M*_mix_ is the molar mass of the gas mixture calculated from the molar portions given in the certificate of the primary reference material and the molar masses of CO_2_ and Ar, respectively. *R* represents the universal gas constant with about 8.3144 J mol^-1^ K^-1^ and for the temperature *T* in K, a value of 293.15 K was used.

$\rho_{mix}=\frac{P\cdot M_{mix}}{R\cdot T}$ Eq. S1


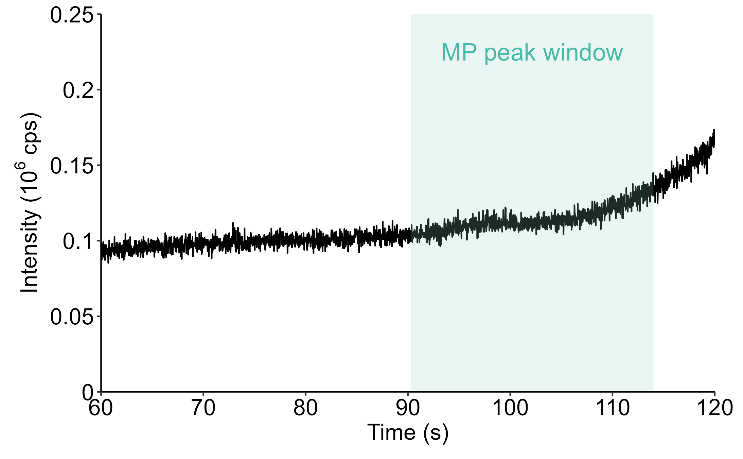


Figure S1: ^13^C^+^ time scan of the ETV/ICP-MS measurement of an empty boat.


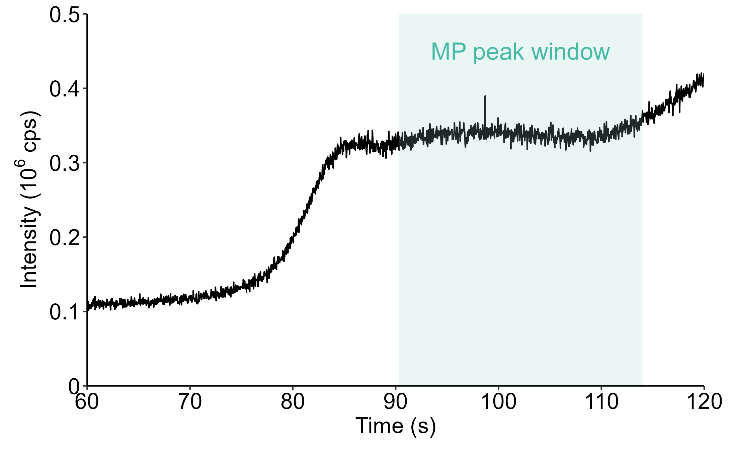


Figure S2: ^13^C^+^ time scan of the ETV/ICP-MS measurement of soil matrix without added MPs.
